# Antitumor activity of gemcitabine against high-grade meningioma *in vitro* and *in vivo*

**DOI:** 10.18632/oncotarget.18827

**Published:** 2017-06-29

**Authors:** Hiroyuki Takeda, Masashi Okada, Kenta Kuramoto, Shuhei Suzuki, Hirotsugu Sakaki, Tomomi Sanomachi, Shizuka Seino, Takashi Yoshioka, Hirofumi Hirano, Kazunori Arita, Chifumi Kitanaka

**Affiliations:** ^1^ Department of Molecular Cancer Science, Yamagata University School of Medicine, Yamagata, Japan; ^2^ Department of Clinical Oncology, Yamagata University School of Medicine, Yamagata, Japan; ^3^ Department of Obstetrics and Gynecology, Yamagata University School of Medicine, Yamagata, Japan; ^4^ Research Institute for Promotion of Medical Sciences, Yamagata University Faculty of Medicine, Yamagata, Japan; ^5^ Department of Neurosurgery, Graduate School of Medical and Dental Sciences, Kagoshima University, Kagoshima, Japan

**Keywords:** cancer, intracranial neoplasm, brain tumor, anaplastic meningioma, malignant meningioma

## Abstract

Currently, there is no established therapeutic option for high-grade meningioma recurring after surgery and radiotherapy, and few chemotherapeutic agents are in development for the treatment of high-grade meningioma. Here in this study, we screened a panel of chemotherapeutic agents for their possible antitumor activity in high-grade meningioma and discovered that high-grade meningioma cells show a preferential sensitivity to antimetabolites, in particular, to gemcitabine. *In vitro*, gemcitabine inhibited the growth of high-grade meningioma cells effectively by inducing S-phase arrest and apoptotic cell death. *In vivo*, systemic gemcitabine chemotherapy suppressed not only tumor initiation but also inhibited the growth and achieved a long-term control of established tumors in xenograft models of high-grade meningioma. Histological analysis indicated that systemic gemcitabine blocks cell cycle progression and promotes apoptotic cell death in tumor cells *in vivo*. Together, our data demonstrate that gemcitabine exerts potent antitumor activity against high-grade meningioma through cytostatic and cytotoxic mechanisms. We therefore propose gemcitabine is a promising chemotherapeutic agent that warrants further investigation as a treatment option for high-grade meningioma.

## INTRODUCTION

Meningiomas, neoplasms derived from arachnoidal (meningothelial) cells [1], comprise the most common primary intracranial tumor [2] and are categorized into three grades based on the World Health Organization (WHO) classification [3]. Whereas Grade I meningiomas are essentially benign and curable by surgical resection alone in the majority of cases, high-grade meningiomas such as Grade II (atypical) and Grade III (anaplastic/malignant) meningiomas are characterized by their aggressive nature and high rate of recurrence, often necessitating radiation-based intervention and systemic chemotherapy for their treatment [4, 5]. Among high-grade meningiomas, surgery- and radiation-refractory recurrent meningiomas in particular have a highly dismal prognosis with the progression-free survival at 6 months reportedly being 26% [6], underscoring the need for effective systemic therapy to treat such recurrent high-grade meningiomas [7, 8]. The guidelines published by the National Comprehensive Cancer Network in 2011 suggested three drugs (hydroxyurea, interferon-alpha, and somatostatin analogues) as treatment options for recurrent meningioma [9]. Nevertheless, the efficacy of these drugs in the treatment of high-grade meningioma is limited or still remains controversial since the guidelines were made based on inevitably limited literature [7, 10], and there is so far no chemotherapeutic agent recommended for the treatment of high-grade meningioma [11]. A number of new drug candidates including cytotoxic, hormonal, and molecular targeting agents, therefore, are being explored currently at the preclinical and clinical levels for systemic chemotherapy of meningioma [6, 7, 10, 12, 13]. However, though sunitinib recently showed promising results in a prospective, single-arm phase 2 trial conducted on recurrent high-grade meningioma cases [14, 15], the clinical benefit of such drugs has yet to be demonstrated conclusively [6, 7, 10, 12, 13]. Apparently, further pursuit of novel drug candidates is required to achieve a better management of therapy-refractory high-grade meningioma.

Gemcitabine is a nucleoside analog that has been used as a chemotherapeutic agent for pancreatic cancer and a variety of other solid tumors including breast, ovarian, and non-small cell lung cancers [16], the efficacy of which in meningioma, however, remains unknown so far. Here in this study, we successfully identified gemcitabine from among a variety of anticancer agents as a promising candidate for the treatment of high-grade meningioma. We show *in vitro* that cell lines derived from high-grade meningiomas are more or as sensitive as gemcitabine-sensitive cell lines derived from human cancers for which gemcitabine is clinically used in the treatment. Furthermore, we also demonstrate systemic gemcitabine treatment effectively suppresses the development and growth of meningioma xenografts *in vivo*. Our data thus point to a possible role of gemcitabine in the management of high-grade meningioma.

## RESULTS

### Anticancer activity of gemcitabine in high-grade meningioma cells *in vitro*

**Figure 1:**
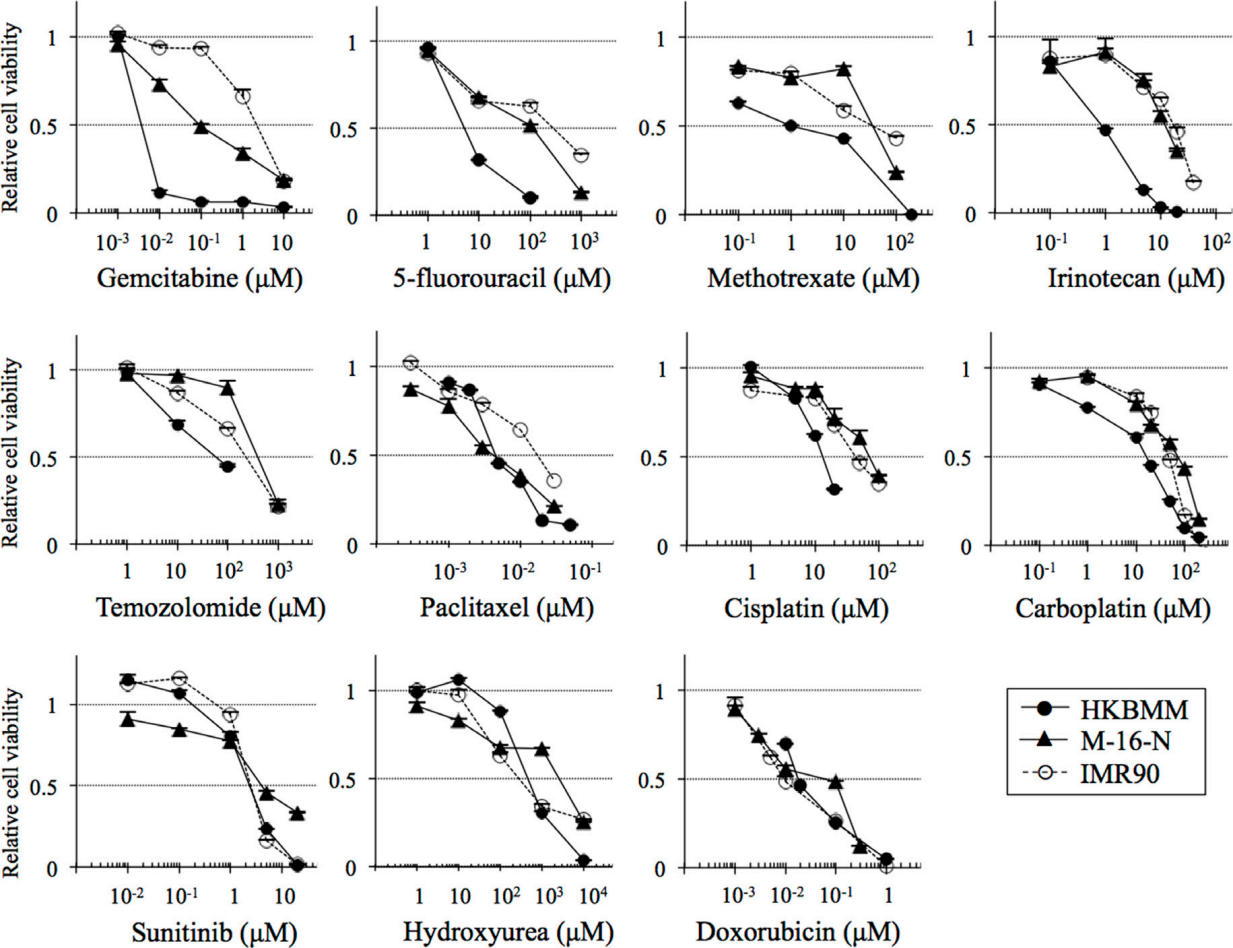
Growth-inhibitory effects of chemotherapeutic agents on high-grade meningioma cells *in vitro* High-grade meningioma cells (HKBMM and M-16-N) and IMR90 normal human fibroblasts were treated with the indicated chemotherapeutic agents for 3 days, and their viability relative to control (cells treated in the absence of the test drug) was determined. Values in the graphs represent means + SD from triplicate samples of a representative experiment repeated with similar results.

To identify candidate chemotherapeutic agents that might be, alone or in combination, of benefit in the treatment of high-grade meningioma, we evaluated drugs of interest, including those that have been tested in the literature, for anticancer activity in high-grade meningioma cells. To this end, we first examined the growth inhibitory effect of each drug on HKBMM malignant meningioma cells in comparison with IMR90 normal human fibroblasts. The results showed that the cell viability curves of HKBMM and IMR90 overlapped for most of the drugs tested including sunitinib and hydroxyurea (Figure 1), with the IC_50_ values of HKBMM for these drugs relative to those of IMR90 being nearly or over 100% (Table 1). On the other hand, we found that HKBMM cells were sensitive to the growth-inhibitory effect of antimetabolites such as gemcitabine, 5-fluorouracil, and methotrexate. In particular, HKBMM cells were exquisitely more sensitive to gemcitabine compared with normal fibroblasts, with the relative IC_50_ value as low as 0.17%. To determine whether this specific sensitivity to gemcitabine is unique to HKBMM cells or is shared by other high-grade meningioma cells, we conducted the same experiments using M-16-N, a primary culture of high-grade meningioma cells established directly from surgical samples resected from a patient with atypical meningioma. Significantly, M-16-N cells showed a similar sensitivity pattern to HKBMM cells in that M-16-N cells were resistant to drugs to which HKBMM cells showed resistance and vice versa (Table 1). And again, M-16-N cells were by far more sensitive to gemcitabine than the other drugs just as HKBMM cells were. Thus, the results suggest that high-grade meningioma cells may be sensitive to gemcitabine among other chemotherapeutic agents.

**Table 1:** The relative and absolute IC_50_ values of the chemotherapeutic agents tested in this study

	IC_50_ (μM)			[A]/[C] × 100 (%)	[B]/[C] × 100 (%)
	HKBMM [A]	M-16-N [B]	IMR90 [C]		
Gemcitabine	0.00365	0.0888	2.15	0.170	4.12
5-fluorouracil	5.17	110	281	1.84	39.2
Methotrexate	0.979	35.4	34.5	2.84	103
Irinotecan	0.827	11.9	17.0	4.87	70.0
Temozolomide	58.2	396	230	25.3	172
Paclitaxel	0.00450	0.00421	0.0173	26.0	24.4
Cisplatin	13.0	70.2	43.1	30.3	163
Carboplatin	17.9	71.7	46.4	38.6	155
Sunitinib	2.36	4.03	2.48	95.0	162
Hydroxyurea	460	2580	279	165	924
Doxorubicin	0.0179	0.0598	0.00928	193	644

**Figure 2:**
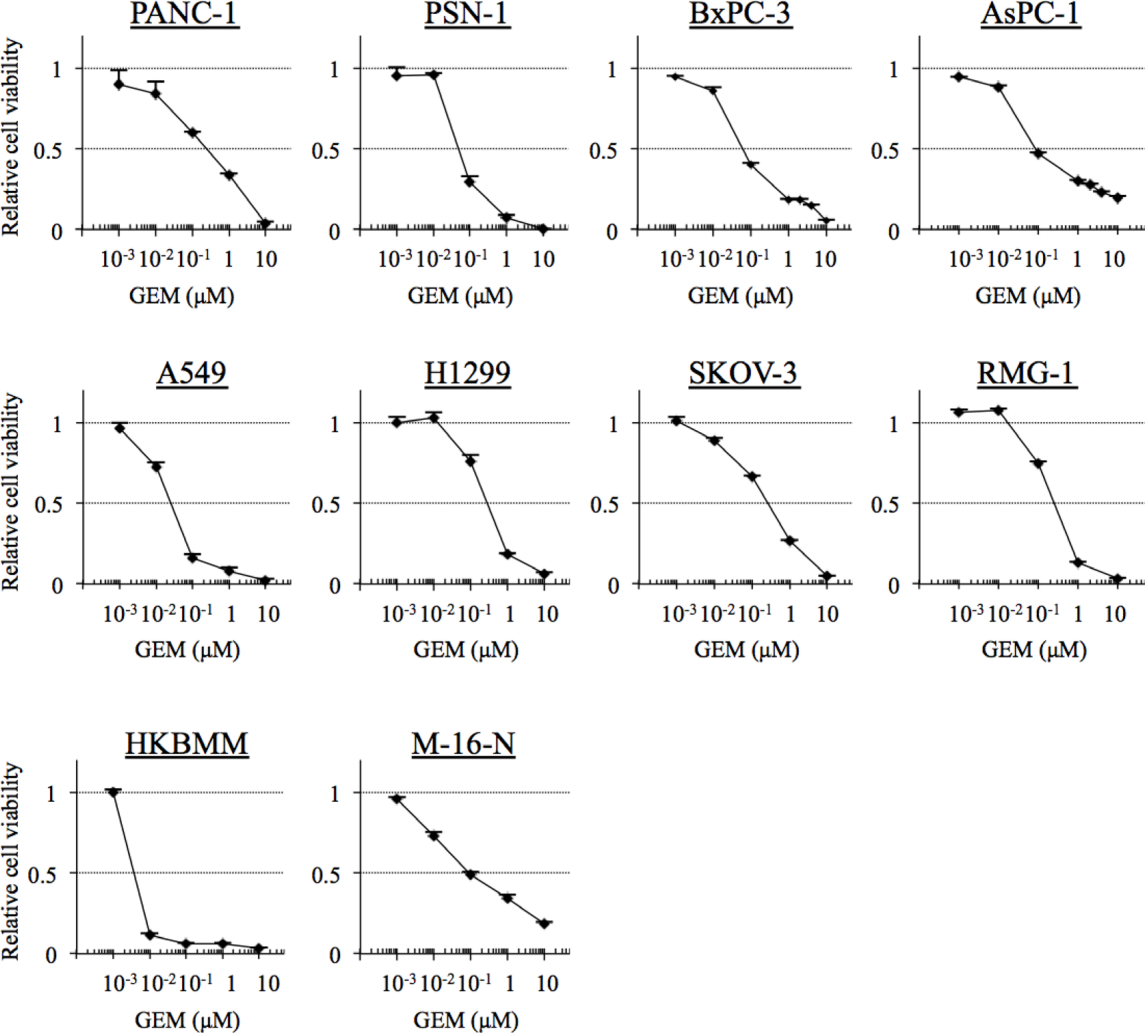
The gemcitabine sensitivity of cell lines derived from cancers for the treatment of which gemcitabine is indicated Pancreatic cancer cells (PANC-1, PSN-1, BxPC-1 and AsPC-1), non-small cell lung cancer cells (A549 and H1299), ovarian cancer cells (SKOV-3 and RMG-1), and high-grade meningioma cells (HKBMM and M-16-N) were treated with the indicated concentrations of gemcitabine (GEM) for 3 days, and their viability relative to control (cells treated in the absence of gemcitabine) was determined. Values in the graphs represent means + SD from triplicate samples of a representative experiment repeated with similar results. Note that the data for HKBMM and M-16-N, which were included in this figure for comparison, are identical with those in Figure 1.

Given the possibility that high-grade meningioma cells may have in common relatively high sensitivity to gemcitabine compared with other chemotherapeutic agents, we next asked whether or not the level of sensitivity falls within the clinically significant range. To this end, we compared the gemcitabine sensitivity of high-grade meningioma cells with those of cell lines derived from pancreatic, lung, and ovarian cancers, for the treatment of which gemcitabine is routinely used in the clinical setting [17]. Strikingly, HKBMM and M-16-N cells were more sensitive than PANC-1, a pancreatic cancer cell line known to be gemcitabine-sensitive [18], and were more or as sensitive to gemcitabine as the other cell lines from pancreatic, lung, and ovarian cancers tested in this study (Figure 2 and Table 2). Thus, the results suggest that the gemcitabine sensitivity of high-grade meningioma cells may be of clinical significance.

**Table 2:** Comparison of the IC_50_ values of gemcitabine against high-grade meningioma cells with those against pancreatic, ovarian, and non-small cell lung cancer cell lines

	High-grade meningioma		Pancreatic cancer				Non-small cell lung cancer		Ovarian cancer	
	HKBMM	M-16-N	PANC-1	PSN-1	BxPC-3	AsPC-1	A549	H1299	SKOV-3	RMG-1
IC_50_ (μM)	0.00365	0.0888	0.238	0.0491	0.0602	0.0843	0.0248	0.281	0.256	0.252

**Figure 3:**
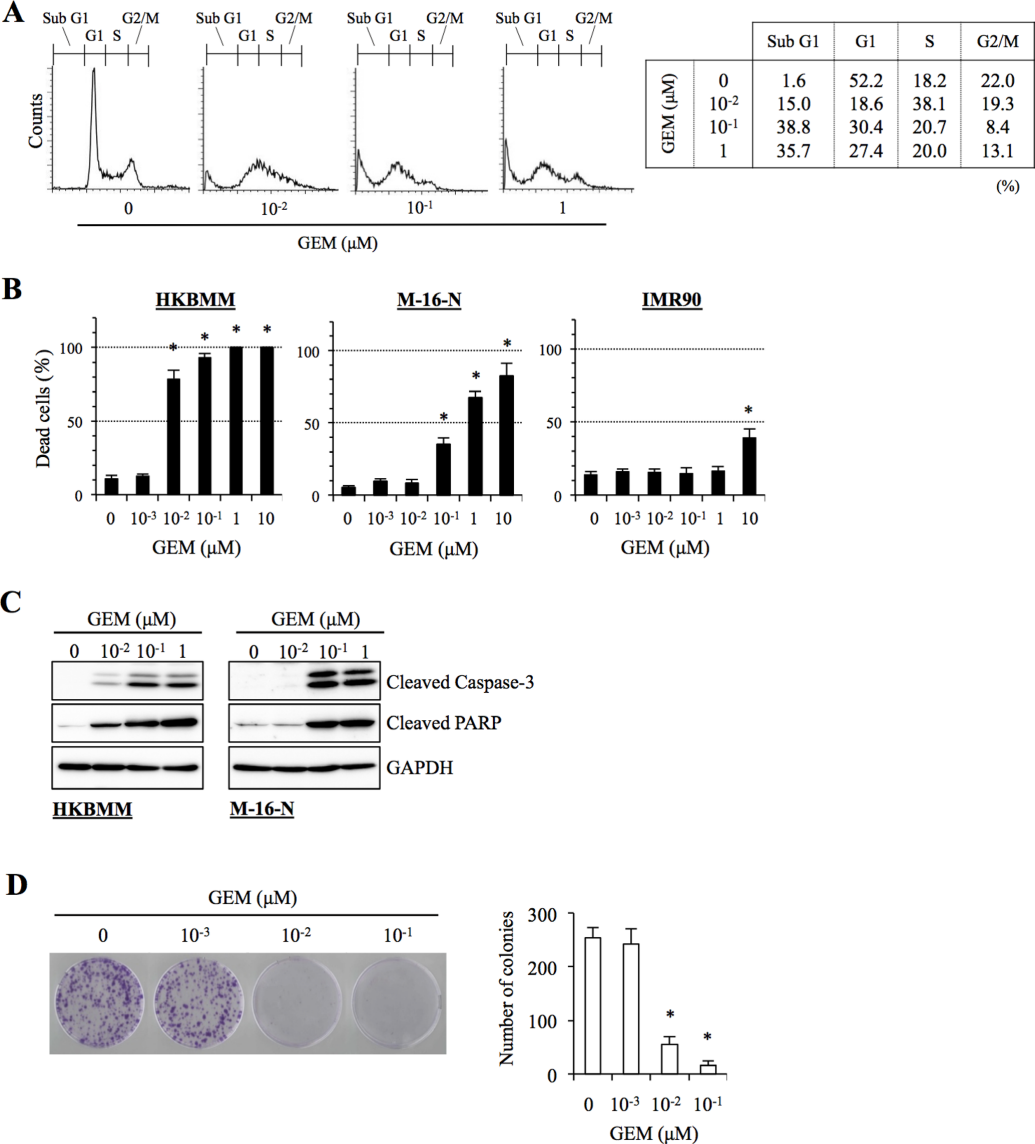
Anti-proliferative and pro-apoptotic activities of gemcitabine in high-grade meningioma cells (**A**) HKBMM cells treated with the indicated concentrations of gemcitabine (GEM) for 24 h were subjected to cell cycle analysis by flow cytometry. Representative flow cytometry histograms are shown, with the percentage of cells in each cell cycle phase tabulated on the right. (**B**) Cells treated with the indicated concentrations of GEM for 3 days were subjected to cell death assay. The graphs show the percentage of dead cells, and values in the graphs represent means + SD from triplicate samples of a representative experiment repeated with similar results. *P < 0.05. (**C**) Cells treated with the indicated concentrations of GEM for 24 h were subjected to immunoblot analysis of cleaved caspase-3 and PARP expression. (**D**) HKBMM cells treated with the indicated concentrations of GEM for 3 days were cultured for another 1 week in the absence of GEM for colony formation assay. An image of a representative experiment (left panel) and the number of colonies (right graph) are shown. Values in the graph represent means + SD from triplicate samples of a representative experiment repeated with similar results. *P < 0.05.

We next determined the mechanism by which gemcitabine inhibits the growth of high-grade meningioma cells. Cell cycle analysis revealed that the proportion of HKBMM cells in the S phase was increased after gemcitabine treatment at 0.01 μM (Figure 3A), implying that S-phase arrest may be a mechanism of gemcitabine-induced growth inhibition. Since the results of the cell cycle analysis also showed an increase in the sub-G1 population when HKBMM cells were exposed to gemcitabine (Figure 3A), we examined whether gemcitabine induces apoptotic cell death in high-grade meningioma cells. Gemcitabine treatment induced cell death in HKBMM and M-16-N cells in a concentration-dependent manner (Figure 3B), which was paralleled by the activation of the caspase-dependent apoptotic program as documented by the cleavage of caspase 3 and PARP (Figure 3C). Thus, the results suggested that gemcitabine may inhibit the growth of high-grade meningioma cells by inducing S-phase arrest and apoptotic cell death.

So far, all the assays in this study were done within three days after the cells were exposed to gemcitabine. To determine whether the growth-inhibitory effect of gemcitabine on high-grade meningioma cells observed in such short-term assays actually translate into long-term inhibition of their clonogenic survival, we conducted a colony formation assay. Since M-16-N cells did not form colonies with a clear margin because of their highly motile nature, the assay was done using HKBMM cells. When cells were treated with gemcitabine for 3 days and then allowed to grow for additional one week in the absence of the drug, colony formation was quite effectively inhibited by gemcitabine as low as 0.01 μM (Figure 3D).

### *In vivo* antitumor activity of systemic gemcitabine in xenograft models of high-grade meningioma

**Figure 4:**
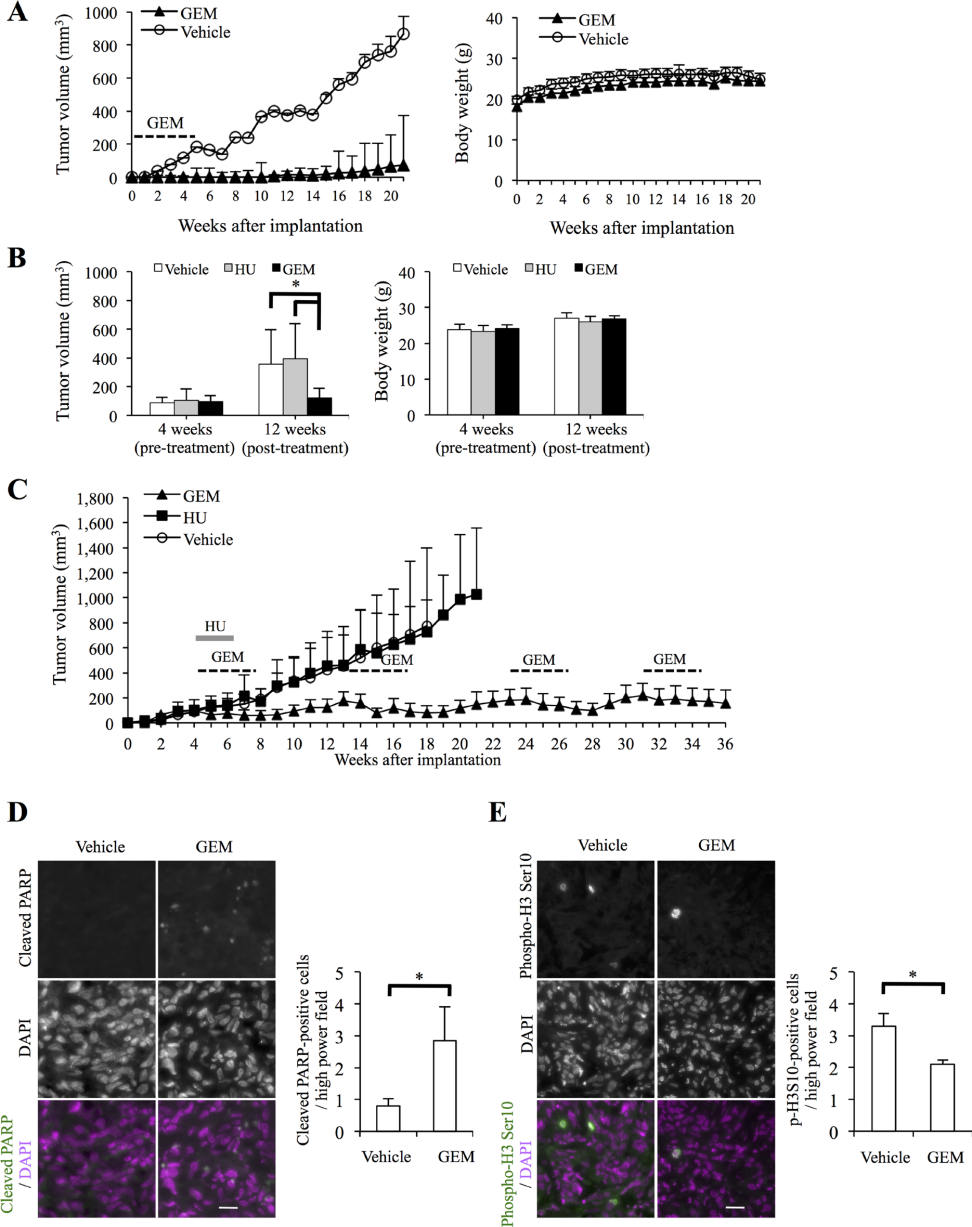
Systemic gemcitabine administration inhibits tumor initiation and progression of high-grade meningioma and provides long-term control (**A**) Mice (three for each group) implanted subcutaneously with 5 × 10^5^ viable HKBMM cells were treated, after randomization according to body weight, with intraperitoneal injection of control vehicle or gemcitabine (GEM, 20 mg/kg twice a week for 4 weeks) starting on the next day of implantation. Tumor volume (left) and the body weight of the mice (right) were measured at the indicated time points. Values in the graphs represent means + SD of each treatment group. (**B**) Mice (three for each group) were implanted subcutaneously with HKBMM cells (bilaterally, 1 × 10^6^ viable cells each) and, after randomization according to tumor volume and body weight, received intraperitoneal injection of vehicle alone, hydroxyurea (HU, 500 mg/kg/day for 15 days) or GEM (20 mg/kg twice a week for 4 weeks), which started at 4 weeks after implantation when the volume of the subcutaneous tumors reached ∼100 mm^3^. Tumor volume (left) and the body weight of the mice (right) measured at 4 (pre-treatment) and 12 (post-treatment) weeks after implantation are shown. Values in the graphs represent means + SD of each treatment group. *P < 0.05. (**C**) The volume of the tumors in the mice described in (B) was subsequently monitored at a weekly interval. Mice in the GEM treatment group were treated repeatedly with the same GEM regimen as indicated in the graph. Values in the graph represent means + SD of each treatment group. (**D**, **E**) Xenograft tumors (∼300 mm^3^) formed by subcutaneous (and bilateral) implantation of HKBMM cells were treated, after randomization according to tumor volume, with systemic administration of the control vehicle or GEM (20 mg/kg twice a week) for 2 weeks (two mice were treated for each group). On the next day of the final administration, the tumors were excised and subjected to immunofluorescence analysis of cleaved PARP (D) and phospho-histone H3 Ser10 (E) expression. Cell nuclei were stained with 4′,6-diamidino-2-phenylindole (DAPI). Representative images (left, scale bars = 20 μm) and graphs indicating the number of positive cells per high-power field (right) are shown. Values in the graphs represent means + SD of three sections from tumors treated as indicated (two tumors from a mouse harboring the largest tumors in each treatment group were analyzed). *P < 0.05.

Having shown that gemcitabine effectively inhibits the clonogenic survival of high-grade meningioma cells and that its growth-inhibitory effect could therefore have therapeutic relevance, we next evaluated the therapeutic potential of systemic gemcitabine administration *in vivo* in meningioma xenograft models. First, we tested the effect of gemcitabine on tumor initiation. To this end, we implanted HKBMM cells into nude mice and initiated on the next day of implantation systemic administration of gemcitabine (20 mg/kg injected intraperitoneally, twice a week for four weeks), a regimen that was well tolerated by recipient mice (Figure 4A, right panel). Whereas tumors developed in all vehicle-treated control mice, tumor development was remarkably delayed or even prevented in mice treated with gemcitabine (Figure 4A, left panel and Supplementary Table 1), demonstrating that gemcitabine inhibits tumor initiation by high-grade meningioma cells *in vivo*. Next, to evaluate the antitumor activity of gemcitabine against established tumors, HKBMM cells were implanted into mice and were allowed to grow for 4 weeks to reach the volume of ∼ 100 mm^3^. We then treated the tumor-bearing mice with the same gemcitabine regimen as above for 4 weeks (i.e., from the 5th week to the 8th week after implantation), while control mice were either treated with hydroxyurea or vehicle alone. At 12 weeks (after implantation), gemcitabine-treated tumors were significantly smaller than control tumors without significant differences in the body weights between the control- and gemcitabine-treated mice (Figure 4B), suggesting that gemcitabine inhibits not only tumor initiation but also the growth of established tumors effectively. Since gemcitabine-treated tumors showed signs of regrowth at 13 weeks (Figure 4C and Supplementary Table 2), we treated them again with the same regimen (from the 13th week to the 16th week). Strikingly, all of the tumors that had started to regrow after the first cycle of gemcitabine treatment shrank after the second cycle of gemcitabine treatment. We therefore treated the gemcitabine-treated tumors similarly thereafter when they showed signs of regrowth; each time the tumors were treated, they responded and began to shrink (Figure 4C and Supplementary Table 2). As a result, whereas all but one of the control tumors grew progressively and became lethal by 35 weeks, all the gemcitabine-treated tumors were successfully kept under control at 36 weeks. In parallel, we also conducted immunofluorescence analysis of established tumors that were resected after being treated with gemcitabine for two weeks. The results demonstrated that apoptotic cells positive for cleaved PARP and mitotic cells with histone H3 phosphorylated on Ser10 [19, 20] were increased and decreased, respectively, in gemcitabine-treated tumors compared to control-treated tumors (Figure 4D and 4E), in support of the idea that gemcitabine induces apoptosis and S-phase arrest in high-grade meningioma cells *in vivo* as well as *in vitro*. Together, the data suggest that gemcitabine exerts potent antitumor activity in xenograft models of high-grade meningioma through cytostatic and cytotoxic mechanisms.

## DISCUSSION

The prognosis of high-grade meningiomas recurring after surgery and radiotherapy is highly dismal, underscoring the dire need to develop chemotherapeutic agents to treat recurrent high-grade meningioma [7, 8]. However, so far, only a limited number of drugs have been shown to be effective against high-grade meningioma in clinical or preclinical animal studies [14, 21–24]. Here in this study, we identified gemcitabine as a promising chemotherapeutic agent against high-grade meningioma. Whereas the growth of high-grade meningioma cells and normal fibroblasts was inhibited comparably by most of the chemotherapeutic agents tested in this study, gemcitabine displayed selective and potent growth-inhibitory activity against high-grade meningioma cells. Furthermore, high-grade meningioma cells were similarly (M-16-N cells) or much more (HKBMM cells) sensitive to gemcitabine than various cell lines derived from cancers for which the clinical benefit of gemcitabine treatment has been well documented, suggesting that gemcitabine would be effective in the treatment of high-grade meningioma similarly to those cancers. Of note, we observed no selective growth inhibitory effect of sunitinib on high-grade meningioma cells over normal fibroblasts, with the IC_50_ being 2.36 μM for HKBMM cells, 4.03 μM for M-16-N cells, and 2.48 μM for IMR90 cells. Significantly, these IC_50_ values are quite comparable with those of sunitinib for high-grade meningioma cells reported previously [25]. Given the therapeutic benefit of sunitinib demonstrated clinically [14], our results suggest sunitinib may either act primarily on tumor endothelium rather than on tumor cells as previously proposed [26] or have a growth inhibitory effect specifically on meningioma cells growing *in vivo*.

In good accordance with the *in vitro* data, the results of the *in vivo* animal experiments clearly demonstrated the potent antitumor activity of gemcitabine in xenograft models of high-grade meningioma. Not only did gemcitabine prevent tumor formation by high-grade meningioma cells, it effectively curbed the growth of established tumors. Most importantly, the xenograft tumors responded to gemcitabine repeatedly even when gemcitabine was administered at the time of tumor regrowth after each cycle of gemcitabine administration, demonstrating that high-grade meningioma can be treated continually with gemcitabine without eliciting resistance. As a result, the tumors have been successfully controlled for more than half a year after the initiation of gemcitabine treatment. To date, there have been only few xenograft studies of high-grade meningioma in which the effect of chemotherapeutic agents was examined. In those studies, mTORC1 inhibitors [23], Pak inhibitors [21], celecoxib (cox 2 inhibitor) [24], and cerulenin (fatty acid synthase inhibitor) [22] were shown to slow tumor growth significantly compared to their respective control, yet none of the drugs successfully prevented progressive tumor growth. Accordingly, their long-term effects on tumor growth were not investigated, with the longest observation period being less than 2 months. In this regard, gemcitabine is the first-ever chemotherapeutic agent that has been demonstrated to be capable of long-term control of tumor growth in a preclinical animal model of high-grade meningioma. Since gemcitabine has been reported to have an excellent, low toxicity profile suitable for long-term administration [17, 27–30], our results suggest gemcitabine may be highly promising and advantageous in the long-term management of high-grade meningioma. It needs to be emphasized here that the gemcitabine regimen used in our xenograft study (intraperitoneal gemcitabine 20 mg/kg, twice a week for 4 weeks, given at the time of tumor regrowth) was not optimized and was far less intense compared with those (intraperitoneal gemcitabine 100∼125 mg/kg, twice a week continuously) widely used to treat xenograft tumors in nude mice [31, 32], apparently allowing for increased dosing in future experiments. Thus, the modest regimen adopted in this study may account for why complete tumor remissions were not achieved in the established tumor model. Alternatively, it is also conceivable that the population of non-proliferating tumor cells that are therefore insensitive to gemcitabine may have expanded and occupied the majority of the tumor mass as tumors grew.

Gemcitabine has been combined with various chemotherapeutic compounds, among others, cisplatin. *In vitro* studies demonstrated synergistic interactions between gemcitabine and cisplatin, and the combination of these drugs has now become a standard regimen in the treatment of non-small cell lung cancer and bladder cancer [17]. Although high-grade meningioma cells proved to be resistant to cisplatin in our study, it is possible that cisplatin may enhance their sensitivity to gemcitabine and thus justify combination with gemcitabine. Notably, our results suggested that high-grade meningioma cells may be sensitive to 5-fluorouracil and paclitaxel, for which the efficacy of combination with gemcitabine has been documented [33]. Of particular interest, in patients treated with gemcitabine, paclitaxel dose-dependently increased the peak concentration of gemcitabine-5’-triphosphate (dFdCTP) in white blood cells [33]. More recently, it has been demonstrated that nab-paclitaxel in combination with gemcitabine for pancreatic cancer patients increases dFdCTP concentration in tumor tissues and that, as an explanation for this observation, nab-paclitaxel and paclitaxel decrease the protein expression of cytidine deaminase *in vivo* and *in vitro*, respectively [34]. Thus, our results together with these observations warrant future investigations on the antitumor activity of paclitaxel in combination with gemcitabine against high-grade meningioma cells. In addition to the drugs mentioned so far, combining gemcitabine with sunitinib may also be of interest given the results of the recent phase 2 study of sunitinib in the treatment of recurrent high-grade meningioma. In this regard, there is a preclinical study demonstrating the benefit of combining these two drugs in a mouse xenograft model of pancreatic cancer [35]. Although the benefit of adding sunitinib to gemcitabine in the treatment of advanced pancreatic cancer failed to be demonstrated in a phase 2 clinical trial [36], the results of another phase 2 trial recently showed that gemcitabine combined with sunitinib is an active and well-tolerated combination for patients with aggressive renal cell carcinoma [37]. Apparently, gemcitabine combined with sunitinib is an attractive regimen for the treatment of high-grade meningioma that deserves future investigations.

At present, it remains unknown why high-grade meningioma cells are sensitive to gemcitabine, in part because mechanisms that determine cells’ sensitivity and resistance to gemcitabine *per se* are not yet well understood despite its relatively broad and common use. However, accumulating evidence has suggested the importance of enzymes and other molecules involved in nucleotide metabolism in conferring sensitivity and resistance on cells to gemcitabine. Among such enzymes is deoxycytidine kinase (dCK), which catalyzes the rate-limiting phosphorylation reaction essential for gemcitabine, a prodrug, to be metabolized to its active triphosphate form and as such has been demonstrated to be a key determinant of gemcitabine sensitivity *in vitro*, *in vivo*, and in patients [16]. Notably, the protein expression of dCK is under the control of an RNA-binding protein Hu antigen R (HuR) that controls gene expression posttranscriptionally [38]. Treatment of cells with gemcitabine induces translocation of HuR from the nucleus to the cytoplasm and promotes HuR binding to the 3′-untranslated region of dCK mRNA and thereby the protein expression of dCK. Consistent with its role as a modulator of a critical enzyme in gemcitabine metabolism, cytoplasmic HuR expression has also been associated with better response to gemcitabine treatment [38, 39]. Significantly, HuR expression has been detected in meningioma tissues in previous studies, and a very recent study demonstrated that HuR expression, both cytoplasmic and total, increases with the grade of malignancy and is associated with poor prognosis of meningioma [40–42]. Although there is currently no report available that examined the expression of dCK in meningioma, it would be interesting to speculate that HuR overexpression in high-grade meningioma increases gemcitabine sensitivity through increased dCK protein expression and as such may serve as a biomarker with which to predict gemcitabine sensitivity of meningioma. Apparently, the molecular mechanism underlying the sensitivity of high-grade meningioma to gemcitabine, including the role of HuR and dCK expression, is an important topic of future studies.

In conclusion, while the mechanism behind the sensitivity of high-grade meningioma cells to gemcitabine awaits future elucidation, our *in vitro* and *in vivo* data demonstrating the potent growth inhibitory effect of gemcitabine against high-grade meningioma cells provide strong rationale to test its therapeutic efficacy for high-grade meningioma in future preclinical and clinical trials. Gemcitabine alone or in combination with other chemotherapeutic agents might become a promising, viable regimen in the treatment of intractable, recurrent cases of high-grade meningioma for which therapeutic options are highly limited.

## MATERIALS AND METHODS

### Antibodies and reagents

Hydroxyurea was purchased from Tokyo Chemical Industry Co., Ltd. (Tokyo, Japan) and dissolved in distilled water to prepare a 1 M stock solution. Gemcitabine, irinotecan, carboplatin and doxorubicin were purchased from Wako Pure Chemical Industries, Ltd. (Osaka, Japan) and dissolved in dimethylsulfoxide (DMSO) to prepare 1 mM, 20 mM, 25 mM and 10 mM stock solutions, respectively. Methotrexate was also purchased from Wako and dissolved in 1 M NaOH to prepare a 10 mM stock solution. Sunitinib, 5-fluorouracil, paclitaxel and cisplatin were purchased from Sigma (St. Louis, MO, USA) and dissolved in DMSO to prepare 10 mM, 10 mM, 1 mM and 100 mM stock solutions, respectively. Temozolomide was purchased from LKT Laboratories, Inc. (St. Paul, MN, USA) and dissolved in DMSO to prepare a 50 mM stock solution. Antibodies such as Cleaved Caspase-3 (Asp175, #9661), Cleaved PARP (Asp214, #9541), Merlin (#12888), Vimentin (#5741), phospho-Histone H3 (S10, #9706), Cleaved PARP (Asp214, Fluorescein conjugate, #9547), and GAPDH (#5174) were purchased from Cell Signaling Technology, Inc. (Danvers, MA, USA).

### Cell culture

HKBMM (human malignant meningioma cell line) was obtained from the Riken BioResource Center (Tsukuba, Japan) and was maintained in Ham’s F12 medium supplemented with 10% fetal bovine serum (FBS). M-16-N was established by H.H. from brain tumor tissue of recurrent atypical meningioma removed from a 54-year-old female, non-neurofibromatosis patient who had been treated for intracranial recurrence (by surgery and radiosurgery) and extracranial metastasis (left lung, surgically removed) of meningioma. In brief, the tumor tissue was rinsed in phosphate-buffered saline (PBS) and then minced in a culture dish with RPMI-1640 medium supplemented with 10% FBS. Three days later, adherent cells were dissociated and re-plated as passage 1 of M-16-N, which was propagated in RPMI-1640 medium supplemented with 10% FBS up to passage 4 and with 20% FBS thereafter. In principle, low passage number (< 10) M-16-N cells were used in this study. Immunoblot analysis confirmed that both HKBMM and M-16-N were positive for vimentin but that the latter (M-16-N) was negative for merlin, the NF2 gene product, whereas the former (HKBMM) was positive (Supplementary Figure 1). PANC-1 human pancreatic cancer cell line was obtained from Cell Resource Center for Biomedical Research, Institute of Development, Aging and Cancer, Tohoku University. PSN-1 was a kind gift from Dr. T. Yoshida (National Cancer Center Research Institute, Tokyo, Japan), who originally established the cell line from pancreatic adenocarcinoma tissue [43]. Pancreatic cancer cell lines BxPC-3 and AsPC-1 were also provided by Dr. T. Yoshida. A549 and H1299, human non-small cell lung cancer cell lines, were obtained from the Riken BioResource Center. RMG1 human ovarian cancer cell line was kindly provided by Dr. S. Nozawa and Dr. D. Aoki (Keio University, Japan). These cell lines were maintained either in DMEM/F12 medium (PANC-1, PSN-1, A549, H1299 and RMG-1) or RPMI-1640 medium (BxPC-3 and AsPC-1), supplemented with 10% FBS. SKOV3 human ovarian cancer cell line was purchased from American Type Culture Collection (ATCC, Manassas, VA, USA) and maintained in M199:105 medium, a 1:1 mixture of M199 and MCDB105 media supplemented with 10% FBS. Normal human IMR90 fetal lung fibroblasts were obtained from ATCC and maintained in DMEM supplemented with 10% FBS. All culture media were supplemented with 100 U/mL penicillin and 100 μg/mL streptomycin. The authenticity of cell lines used in this study (HKBMM, PANC-1, PSN-1, A549, H1299, SKOV-3, and RMG-1) was verified by the genotyping of short tandem repeat (STR) loci (BEX CO., LTD, Tokyo, Japan) followed by comparison to the ATCC STR Database for Human Cell lines. All IMR90 experiments were performed using low passage number (< 8) cells.

### Cell viability assay

Cell viability was determined by tetrazolium salt reduction method using WST-8 according to the manufacturer’s instructions (Cell Counting Kit-8, DOJINDO LABORATORIES, Kumamoto, Japan) [44–46]. Briefly, 1,000–2,000 cells/well were plated in 96-well tissue culture plates and, after 24 h, were treated with drugs as described in the figure legends. Then WST-8 reagent was added and the cells were incubated for 1–3 h at 37°C. Absorbance at 450 nm was measured using a spectral scanning multimode plate reader, Valioskan Flash (Thermo Fisher Scientific, Waltham, MA, USA). Relative cell viability was calculated as a percentage of absorbance of treated samples relative to that of controls. To determine the IC_50_ values, we used the following formula as previously reported [47, 48]; IC_50_ = 10^[log(A/B) × (50-C)]/[(D-C) + Log(B)]^ where A and B are the corresponding concentrations of the test drug directly above and below 50% inhibition, respectively, and C and D are the percentage of inhibition directly below and above 50% inhibition, respectively.

### Cell death assay

Viable and dead cells were identified by their ability and inability to exclude vital dyes, respectively [49–51]. Briefly, cells were stained with 0.2% trypan blue for 1 min at room temperature, and the numbers of viable and dead cells was determined under a phase-contrast microscope using a hemocytometer. The percentage of dead cells was defined as 100 × (number of dead cells/[the number of viable + dead cells]).

### Cell cycle analysis

Cell cycle profiles were analyzed by the standard propidium iodide (PI) staining protocol as described previously [46]. In brief, both adherent and non-adherent cells were collected and, after being washed once with PBS, fixed with cold 70% ethanol at –20°C overnight or longer. After centrifugation for 10 min at 1,000 × g, the pellets were washed with PBS twice. The cells were then incubated with PI (20 μg/mL) and RNase A (10 μg/mL) in PBS for 30 min at 37°C in the dark and subjected to flow cytometry analysis on FACSCantoTM II Flow Cytometer (BD Biosciences, Franklin Lakes, NJ, USA). All collected data were analyzed using the FlowJo software, version 7.6.5 (Treestar Inc., Ashland, OR, USA).

### Immunoblot analysis

Immunoblot analysis was conducted as described previously [49, 51–53]. In brief, cells were washed with ice-cold PBS and lysed in RIPA buffer [10 mM Tris-HCl (pH 7.4), 0.1% SDS, 0.1% sodium deoxycholate, 1% NP-40, 150 mM NaCl, 1 mM EDTA, 1.5 mM Na_3_VO_4_ 10 mM NaF, 10 mM sodium pyrophosphate, 10 mM sodium β-glycerophosphate and 1% protease inhibitor cocktail set III (Sigma)]. After centrifugation for 10 min at 14,000 × g at 4°C, the supernatants were recovered as the cell lysates, and the protein concentration of the cell lysates was determined using a BCA protein assay kit (Pierce Biotechnology, Inc., Rockford, IL, USA). Cell lysates containing equal amounts of protein were separated by SDS-PAGE and transferred to a polyvinylidene difluoride membrane. The membrane was probed with a primary antibody and then with an appropriate HRP-conjugated secondary antibody according to the protocol recommended by the manufacturer of each antibody. Immunoreactive bands were visualized using Immobilon Western Chemiluminescent HRP Substrate (Millipore, Billerica, MA, USA).

### Colony formation assay

Colony formation assay was performed as described previously [46, 47, 50]. In brief, cells were seeded at a low, colony-forming density (1,000 cells/60-mm dish) and treated and cultured for approximately 1 week. The cells were then fixed with formaldehyde (4% v/v), followed by staining with crystal violet (0.1% w/v). Colonies (consisting of ≥ 50 cells derived from a single cell) were counted using a microscope.

### Immunofluorescence

Xenograft tumors treated as in the figure legend were removed from the mice and fixed with 4% paraformaldehyde in PBS (pH7.4) for 2 days at 4°C. Each formalin-fixed and paraffin-embedded specimen was cut into 3-μm thick sections, followed by deparaffinization and antigen retrieval using citric acid (Antigen Retrieval Solution pH 6; IATRON LABORATORIES INC., Tokyo, Japan) in an autoclave (2 atmospheres, 121°C, 20 min). Sections were incubated with primary antibodies at 4°C overnight, followed by fluorescein-conjugated AffiniPure donkey anti-mouse IgG (H+L) (Jackson ImmunoResearch Laboratories, West Grove, PA, USA) in case of phospho-histone H3 staining, and observed under a fluorescence microscope (CKX41; OLYMPUS, Tokyo, Japan). Cell nuclei were stained with 4′,6-diamidino-2-phenylindole (DAPI). For quantitative analysis, at least 10 high-power fields were assessed for each section.

### Mouse studies

Mouse xenograft studies were carried out essentially as previously described [49, 52, 53]. In brief, 6- to 9-week-old male BALB/cAJcl-nu/nu mice (CLEA Japan Inc., Tokyo, Japan) were implanted subcutaneously in the flank region with cells suspended in 200 μL of sterilized PBS under avertin (0.375 g/kg intraperitoneally) anesthesia. After implantation, the recipient mice were monitored for general health status and presence of subcutaneous tumors. Tumor volume was determined by measuring tumor diameters (measurement of 2 perpendicular axes of tumors) using a caliper and calculated as 1/2 × (larger diameter) × (smaller diameter)^2^. For systemic administration of gemcitabine and hydroxyurea, gemcitabine (8 mg/mL in DMSO) and hydroxyurea (200 mg/mL in distilled water) stock solutions were diluted in PBS to prepare 200 μL solutions for each injection. The gemcitabine and hydroxyurea solutions were injected intraperitoneally into nude mice. All vehicle- and gemcitabine-treated mice received an equal volume of DMSO per body weight (3.6 mL/kg). All animal experiments were performed under a protocol approved by the Animal Research Committee of Yamagata University.

### Statistical analysis

Results are expressed as the mean + standard deviation (SD), and differences were compared using the two-tailed Student’s *t*-test. *P*-values < 0.05 were considered statistically significant and are indicated with asterisks in the figures.

## SUPPLEMENTARY MATERIALS FIGURE AND TABLES




