# Supplementary material for: Antitumor activity of gemcitabine against high-grade meningioma in vitro and in vivo
Source: Oncotarget. 2017 Jun 29;8(53):90996–1008. doi: 10.18632/oncotarget.18827 (PMC5710900; doi:10.18632/oncotarget.18827)
Supplement: Supplementary file 1 [file oncotarget-08-90996-s001.pdf]

Antitumor activity of gemcitabine against high-grade meningioma *in vitro* and *in vivo*

Supplementary Materials

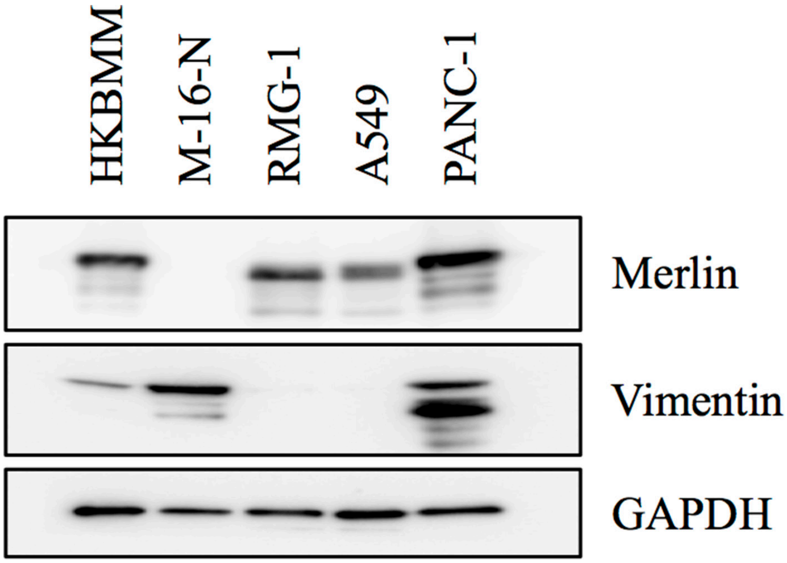

Supplementary Figure 1: Expression of merlin and vimentin in high-grade meningioma cells and in cell lines derived from other types of cancer. Cell lysates prepared from HKBMM and M-16-N (high-grade meningioma), RMG-1 (ovarian cancer), A549 (non-small cell lung cancer), PANC-1 (pancreatic cancer) cells maintained under their standard culture conditions were subjected to immunoblot analysis of the endogenous expression of merlin and vimentin.

Supplementary Table 1: The effect on tumor growth of gemcitabine treatment immediately following tumor cell implantation

weeks	0	1	2	3	4	5	6	7	8	9	10	11	12	13	14	15	16	17	18	19	20	21
GEM	0	0	0	0	0	0	0	0	0	0	0	18.0	32.0	32.0	18.0	6.0	4.0	4.0	6.0	0	1.0	0
	0	0	0	0	0	0	0	0	0	0	0	0	13.5	13.5	13.5	48.0	75.0	87.5	100.0	144.0	196.0	220.5
	0	0	0	0	0	0	0	0	0	0	0	0	0	0	0	0	0	0	0	0	0	0
Vehicle	0	0	48.0	75.0	112.5	245.0	245.0	180.0	269.5	294.0	486.0	384.0	352.0	352.0	320.0	550.0	550.0	726.0	936.0	936.0	1008.0	1267.5
	0	0	40.0	87.5	126.0	196.0	126.0	126.0	256.0	196.0	288.0	405.0	405.0	405.0	405.0	445.5	405.0	550.0	600.0	726.0	726.0	786.5
	0	0	22.5	64.0	108.0	108.0	126.0	108.0	196.0	220.5	320.0	405.0	364.5	445.5	405.0	445.5	726.0	500.0	550.0	550.0	550.0	550.0

Supplementary Table 2: The effect of repeated cycles of gemcitabine treatment on the growth of established tumors. See Supplementary_Table_2
