# Supplementary material for: Antitumor activity of gemcitabine against high-grade meningioma in vitro and in vivo
Source: Oncotarget. 2017 Jun 29;8(53):90996–1008. doi: 10.18632/oncotarget.18827 (PMC5710900; doi:10.18632/oncotarget.18827)
Supplement: Supplementary file 2 [file oncotarget-08-90996-s002.docx]

Supplementary Table 2

|  |  |  |  |  |  | Gemcitabine | | | |  |  |  |  |  | Gemcitabine | | | |  | |  | |  | |  | |  |
| --- | --- | --- | --- | --- | --- | --- | --- | --- | --- | --- | --- | --- | --- | --- | --- | --- | --- | --- | --- | --- | --- | --- | --- | --- | --- | --- | --- |
|  | weeks | 0 | 1 | 2 | 3 | 4 | 5 | 6 | 7 | 8 | 9 | 10 | 11 | 12 | 13 | 14 | 15 | 16 | | 17 | | 18 | | 19 | | 20 | |
| GEM | R | 0 | 0 | 75.0 | 162.0 | 144.0 | 62.5 | 108.0 | 87.5 | 126.0 | 75.0 | 75.0 | 126.0 | 126.0 | 269.5 | 294.0 | 125.0 | 245.0 | | 220.5 | | 162.0 | | 162.0 | | 245.0 | |
|  | L | 0 | 0 | 64.0 | 87.5 | 144.0 | 112.5 | 72.0 | 126.0 | 87.5 | 72.0 | 125.0 | 112.5 | 125.0 | 196.0 | 162.0 | 87.5 | 144.0 | | 87.5 | | 144.0 | | 144.0 | | 100.0 | |
|  | R | 0 | 0 | 64.0 | 126.0 | 108.0 | 27.0 | 48.0 | 4.0 | 40.0 | 22.5 | 56.0 | 56.0 | 56.0 | 144.0 | 144.0 | 87.5 | 108.0 | | 75.0 | | 75.0 | | 62.5 | | 75.0 | |
|  | L | 0 | 0 | 48.0 | 64.0 | 80.0 | 27.0 | 22.5 | 18.0 | 0.0 | 0.0 | 22.5 | 48.0 | 48.0 | 48.0 | 180.0 | 112.5 | 162.0 | | 100.0 | | 64.0 | | 87.5 | | 144.0 | |
|  | R | 0 | 0 | 40.0 | 56.0 | 72.0 | 87.5 | 75.0 | 62.5 | 40.0 | 100.0 | 144.0 | 245.0 | 245.0 | 245.0 | 100.0 | 27.0 | 31.5 | | 22.5 | | 18.0 | | 40.0 | | 87.5 | |
|  | L | 0 | 0 | 56.0 | 87.5 | 27.0 | 75.0 | 108.0 | 64.0 | 64.0 | 126.0 | 144.0 | 144.0 | 144.0 | 144.0 | 48.0 | 22.5 | 18.0 | | 18.0 | | 18.0 | | 18.0 | | 40.0 | |
|  |  |  |  |  |  |  |  |  |  |  |  |  |  |  |  |  |  |  | |  | |  | |  | |  | |
|  |  |  |  |  |  | Hydroxyurea | |  |  |  |  |  |  |  |  |  |  |  | |  | |  | |  | |  | |
| HU | R | 0 | 22.5 | 100.0 | 75.0 | 144.0 | 144.0 | 196.0 | 220.5 | 220.5 | 288.0 | 352.0 | 352.0 | 500.0 | 486.0 | 600.0 | 600.0 | 650.0 | | 650.0 | | 650.0 | | 650.0 | | 526.5 | |
|  | L | 0 | 32.0 | 48.0 | 245.0 | 245.0 | 269.5 | 269.5 | 526.5 | 269.5 | 650.0 | 700.0 | 847.0 | 907.5 | 907.5 | 1152.0 | 1152.0 | 968.0 | | 1080.0 | | 1008.0 | | 1080.0 | | 1800.0 | |
|  | R | 0 | 18.0 | 75.0 | 126.0 | 144.0 | 196.0 | 245.0 | 320.0 | 320.0 | 486.0 | 384.0 | 567.0 | 700.0 | 567.0 | 750.0 | 700.0 | 800.0 | | 800.0 | | 750.0 | | 1028.5 | | 1028.5 | |
|  | L | 0 | 18.0 | 0 | 40.0 | 40.0 | 75.0 | 62.5 | 75.0 | 75.0 | 87.5 | 220.5 | 245.0 | 245.0 | 384.0 | 526.5 | 384.0 | 700.0 | | 750.0 | | 1080.0 | | 1352.0 | | 1521.0 | |
|  | R | 0 | 0 | 27.0 | 18.0 | 32.0 | 32.0 | 22.5 | 48.0 | 75.0 | 126.0 | 100.0 | 144.0 | 144.0 | 162.0 | 220.5 | 162.0 | 245.0 | | 245.0 | | 352.0 | | 405.0 | | 445.5 | |
|  | L | 0 | 0 | 0 | 13.5 | 18.0 | 62.5 | 48.0 | 87.5 | 75.0 | 126.0 | 196.0 | 220.5 | 245.0 | 245.0 | 269.5 | 352.0 | 384.0 | | 486.0 | | 526.5 | | 650.0 | | 607.5 | |
|  |  |  |  |  |  |  |  |  |  |  |  |  |  |  |  |  |  |  | |  | |  | |  | |  | |
| Vehicle | R | 0 | 0 | 56.0 | 100.0 | 144.0 | 162.0 | 56.0 | 56.0 | 87.5 | 220.5 | 144.0 | 144.0 | 126.0 | 162.0 | 162.0 | 162.0 | 220.5 | | 220.5 | | 220.5 | | 144.0 | | 171.5 | |
|  | L | 0 | 0 | 56.0 | 75.0 | 87.5 | 171.5 | 108.0 | 87.5 | 100.0 | 87.5 | 75.0 | 40.0 | 108.0 | 40.0 | 108.0 | 75.0 | 126.0 | | 126.0 | | 108.0 | | 126.0 | | 75.0 | |
|  | R | 0 | 0 | 40.0 | 87.5 | 126.0 | 196.0 | 220.5 | 288.0 | 320.0 | 405.0 | 445.5 | 445.5 | 550.0 | 600.0 | 650.0 | 650.0 | 650.0 | | 600.0 | | 650.0 | | 700.0 | | 600.0 | |
|  | L | 0 | 0 | 32.0 | 75.0 | 40.0 | 87.5 | 87.5 | 126.0 | 256.0 | 364.5 | 364.5 | 405.0 | 550.0 | 550.0 | 445.5 | 726.0 | 726.0 | | 726.0 | | 786.5 | | 847.0 | | 847.0 | |
|  | R | 0 | 32.0 | 13.5 | 40.0 | 87.5 | 87.5 | 220.5 | 162.0 | 144.0 | 196.0 | 320.0 | 320.0 | 352.0 | 352.0 | 486.0 | 650.0 | 700.0 | | 650.0 | | 847.0 | | Sacrificed | |  | |
|  | L | 0 | 0 | 0 | 56.0 | 48.0 | 48.0 | 100.0 | 196.0 | 220.5 | 405.0 | 665.5 | 786.5 | 847.0 | 1008.0 | 1267.5 | 1352.0 | 1436.5 | | 1912.5 | | 2025.0 | |  |  |  | |

|  |  |  |  | Gemcitabine | | | |  |  |  |  | Gemcitabine | | | |  |  |
| --- | --- | --- | --- | --- | --- | --- | --- | --- | --- | --- | --- | --- | --- | --- | --- | --- | --- |
|  | weeks | 21 | 22 | 23 | 24 | 25 | 26 | 27 | 28 | 29 | 30 | 31 | 32 | 33 | 34 | 35 | 36 |
| GEM | R | 352.0 | 320.0 | 352.0 | 352.0 | 320.0 | 245.0 | 220.5 | 220.5 | 269.5 | 352.0 | 352.0 | 269.5 | 352.0 | 320.0 | 269.5 | 269.5 |
|  | L | 162.0 | 220.5 | 196.0 | 220.5 | 196.0 | 196.0 | 144.0 | 87.5 | 220.5 | 288.0 | 320.0 | 320.0 | 320.0 | 288.0 | 320.0 | 320.0 |
|  | R | 75.0 | 108.0 | 171.5 | 196.0 | 75.0 | 108.0 | 75.0 | 75.0 | 108.0 | 180.0 | 220.5 | 126.0 | 126.0 | 180.0 | 126.0 | 87.5 |
|  | L | 144.0 | 144.0 | 144.0 | 144.0 | 126.0 | 144.0 | 126.0 | 108.0 | 196.0 | 196.0 | 196.0 | 171.5 | 126.0 | 126.0 | 126.0 | 144.0 |
|  | R | 100.0 | 162.0 | 162.0 | 162.0 | 87.5 | 87.5 | 56.0 | 56.0 | 56.0 | 144.0 | 162.0 | 162.0 | 144.0 | 87.5 | 144.0 | 87.5 |
|  | L | 40.0 | 40.0 | 48.0 | 48.0 | 40.0 | 32.0 | 32.0 | 40.0 | 62.5 | 32.0 | 62.5 | 40.0 | 62.5 | 40.0 | 32.0 | 40.0 |
|  |  |  |  |  |  |  |  |  |  |  |  |  |  |  |  |  |  |
| HU | R | 384.0 | 600.0 | 786.5 | 1098.5 | 907.5 | 1470.0 | 2048.0 | 1568.0 | 2432.0 | 1666.0 | 1666.0 | 1666.0 | Dead |  |  |  |
|  | L | 1080.0 | 1080.0 | 1152.0 | 1436.5 | 1352.0 | 1436.5 | 1436.5 | 1521.0 | 1764.0 | 1764.0 | 1764.0 | 1764.0 |  |  |  |  |
|  | R | 1224.0 | Sacrificed |  |  |  |  |  |  |  |  |  |  |  |  |  |  |
|  | L | 2028.0 |  |  |  |  |  |  |  |  |  |  |  |  |  |  |  |
|  | R | 600.0 | 416.0 | 294.0 | 269.5 | 294.0 | 294.0 | 269.5 | 294.0 | 396.0 | 352.0 | 352.0 | 486.0 | Dead |  |  |  |
|  | L | 847.0 | 1183.0 | 1008.0 | 936.0 | 650.0 | 936.0 | 726.0 | 1008.0 | 1267.5 | 1267.5 | 1267.5 | 1470.0 |  |  |  |  |
|  |  |  |  |  |  |  |  |  |  |  |  |  |  |  |  |  |  |
| Vehicle | R | 144.0 | 87.5 | 75.0 | 40.0 | 75.0 | 126.0 | 40.0 | 75.0 | 40.0 | 40.0 | 32.0 | 32.0 | 32.0 | 32.0 | 32.0 | 32.0 |
|  | L | 87.5 | 48.0 | 40.0 | 75.0 | 40.0 | 62.5 | 40.0 | 40.0 | 32.0 | 13.5 | 13.5 | 32.0 | 32.0 | 13.5 | 13.5 | 32.0 |
|  | R | 567.0 | 526.5 | 700.0 | 800.0 | 800.0 | 750.0 | 800.0 | 800.0 | 850.0 | 1800.0 | 1912.5 | 1512.0 | Sacrificed |  |  |  |
|  | L | 847.0 | 1008.0 | 1008.0 | 1267.5 | 1080.0 | 1080.0 | 1687.5 | 1470.0 | 1800.0 | 1210.0 | 1000.0 | 2890.0 |  |  |  |  |
|  | R |  |  |  |  |  |  |  |  |  |  |  |  |  |  |  |  |
|  | L |  |  |  |  |  |  |  |  |  |  |  |  |  |  |  |  |

**Supplementary Table 2: The effect of repeated cycles of gemcitabine treatment on the growth of established tumors**
